# Mindfulness Affects the Boundaries of Bodily Self-Representation: The Effect of Focused-Attention Meditation in Fading the Boundary of Peripersonal Space

**DOI:** 10.3390/bs14040306

**Published:** 2024-04-09

**Authors:** Salvatore Gaetano Chiarella, Riccardo De Pastina, Antonino Raffone, Luca Simione

**Affiliations:** 1 Institute of Cognitive Sciences and Technologies (ISTC), National Research Council (CNR), 00185 Rome, Italy; 2 International School for Advanced Studies (SISSA), 34136 Trieste, Italy; 3 Department of Psychology, “Sapienza” University of Rome, 00185 Rome, Italy; riccardo.depastina@uniroma1.it (R.D.P.); antonino.raffone@uniroma1.it (A.R.); 4 Dipartimento di Scienze Umanistiche e Sociali Internazionali, UNINT, Università degli Studi Internazionali di Roma, 00147 Rome, Italy

**Keywords:** mindfulness meditation, focused-attention meditation, peripersonal space, bodily self

## Abstract

Peripersonal space (PPS) is a dynamic multisensory representation of the space around the body, influenced by internal and external sensory information. The malleability of PPS boundaries, as evidenced by their expansion after tool use or modulation through social interactions, positions PPS as a crucial element in understanding the subjective experiences of self and otherness. Building on the existing literature highlighting both the cognitive and bodily effects of mindfulness meditation, this study proposes a novel approach by employing focused-attention meditation (FAM) and a multisensory audio–tactile task to assess PPS in both the extension and sharpness of its boundaries. The research hypothesis posits that FAM, which emphasizes heightened attention to bodily sensations and interoception, may reduce the extension of PPS and make its boundaries less sharp. We enrolled 26 non-meditators who underwent a repeated measure design in which they completed the PPS task before and after a 15-min FAM induction. We found a significant reduction in the sharpness of PPS boundaries but no significant reduction in PPS extension. These results provide novel insights into the immediate effects of FAM on PPS, potentially shedding light on the modulation of self–other representations in both cognitive and bodily domains. Indeed, our findings could have implications for understanding the intricate relationship between mindfulness practices and the subjective experience of self within spatial contexts.

## 1. Introduction

Debates in philosophy, psychology, and neuroscience explore how “self” and “others” are represented, acknowledging multifaceted and distinct bodily (minimal self) and cognitive (narrative self) domains [1]. The bodily self, the moment-to-moment phenomenological experience of being the subject of a specific experience, relates to the sense of agency and sense of ownership; conversely, the cognitive or narrative self encompasses the reflective and autobiographical shaping of the self across time [1,2]. While the cognitive self reflects high-ordered neural processes, tied to episodic memory and meta-awareness, the minimal self relies on lower-order brain mechanisms integrating multisensory bodily signals [3]. This low-order sensory integration forms a foundational aspect of bodily self-consciousness, shaping bodily self-identification and the first-person perspective [1,3].

Although the bodily self is strongly related to the body, it has been recently suggested that it may go beyond the physical boundaries of the body, specifically within the functionally reachable space closely surrounding the body, called peripersonal space (PPS) [4]. The PPS is a multisensory representation of the space within which interactions between the body and external (reachable) objects occur. It is the result of a continuous process of integration of internal (e.g., interoceptive, proprioceptive, vestibular) and external (e.g., visual, tactile, auditory) sensory information [5]. Crucially, the boundaries of the PPS are not fixed, but they are shaped by experiences and contexts. For example. they are expanded after active tool use, which allows interactions with non-reachable, far objects, both in monkeys [6] and humans [7], and are modulated by positive or negative social interactions [8]. Given that PPS provides a sensory representation of the space surrounding the body, which separates what is “inside” and what is “outside”, i.e., the near- and far-space [9], it seems to be directly involved in underlying subjective experience and the general representation of the self as distinct from the environment and others [4]. It has been proposed that the multisensory mechanism underlying PPS representation is a key element for the sense of body ownership and bodily self-consciousness, linked to an owned whole body that is spatially located [3,4,10,11,12]. In this regard, evidence of bodily illusions has shown a relationship between the modulation of PPS boundaries and the alterations of the experienced self, suggesting that the experienced self might be constrained by PPS rather than the physical boundaries of the body [13,14,15].

Recent studies have reported that mind–body techniques such as mindfulness meditation (MM) practices can modulate self–other representations. MM is a meditative practice commonly described as paying attention in an open, nonconceptual, and nonjudgmental way, focusing on bodily sensations and mental events with the prospect of cultivating equanimity and awareness [16]. Behavioral and neuroimaging studies showed how MM can shape self and self–other experiences in the cognitive domain, for instance, by intensifying empathy, compassion, and altruism (e.g., [17,18]), reducing self-referential activity [19,20,21,22], and promoting self–other connectedness [23,24,25,26,27,28], providing a promising model for addressing growing public health issues associated with loneliness and isolation [29,30,31]. However, evidence regarding the relationship between mindfulness and the bodily self is much scarcer, with a prevalence of phenomenological studies that reported how mindfulness meditation may alter the perception of the boundaries between the body and the surrounding world [19,24,32]. Mindfulness meditators have reported changes in their sense of self during meditation sessions in terms of (a) diminished sense of boundaries between them and the environment, (b) self-representation extended beyond the body to include stimuli in the external world, and (c) a total disappearing of the boundaries that separate the self vs. non-self. According to Ataria et al. [32], while the perception of physical body boundaries (i.e., the boundaries of the body as an object) remains constant during meditation, participants reported increased flexibility in the sense of boundaries (perceiving the self versus the external world). This flexibility appears to reduce the distinction between “inside” and “outside”, and affects spatial self-location during meditation [32].

On this basis and given that MM mostly emphasizes awareness of internal (bodily sensations and interoception) events, our hypothesis posits that this phenomenological modulation reported between self and others could potentially be related to the modulation of PPS representation, or at least be reflected in such a modulation. Consistent with this perspective, a recent study by Nguyen et al. [33] delved into examining the effect of meditation on PPS. In this study, novice meditators completed an auditory oddball task with the stimuli presented at three different distances from the body: within PPS (near), at the approximate border of PPS (arm-length), and outside of PPS (far). The auditory oddball task was performed before and after a 15-min body-imagery-based meditation, while event-related potentials (ERPs) were recorded as a marker of PPS attentional salience, consistent with Reed et al. [34]. The authors reported that short-term body-imagery-based meditation leads to the shrinking of PPS boundaries (i.e., reduced salience of near-body stimuli, which after meditation are coded as far-body stimuli). The authors argued that meditation reduced the extent of PPS via heightened interoception, according to previous evidence showing a link between increased interoception and PPS shrinkage [35]. While it remains uncertain whether in general the meditation is conclusively linked to heightened interoception (among both experienced and non-experienced meditators; for a review, see [36]), the specific type or nature of the meditation employed complicates the resolution of this conclusion. The participants in this study engaged in the following activities during the 15-min meditation session: 4 min of external (open) monitoring, 2 min of body scan, 2.5 min of focused attention to mood and breath, and finally, 5.5 min of imagery meditation, where they were asked to visualize a light emanating from their bodies and expanding outward in all directions. Therefore, only a small portion of this meditation was directly related to awareness oriented towards bodily sensations and plausibly to interoceptive activities. Moreover, this meditation session was not structured as a typical mindfulness meditation, such as a focused-attention meditation (FAM) or a body-scan meditation. Such typical forms of meditation could be more easily assessed and linked to the existing neuroscientific and clinical literature, e.g., [37,38].

Then, we suggest that the usage of FAM rather than body-imagery-based meditation may be preferable to test the hypothesis of the interoception effect on PPS boundaries. During FAM, participants are guided to focus their attention on a specific object, usually the sensation of breathing, and to sustain it over time [39,40]. Although mind wandering is likely to occur [41], the train of thoughts is not to be judged; rather, in an accepting way, attention needs to be refocused on the sensation of breathing and related bodily experience [16]. Finally, given that mindfulness meditation is a practice focused on the bodily experience, to investigate modulation related to multisensory integration, rather than strictly related to the attentional salience of sensory unimodal stimuli, the multisensory interaction task could be the most adequate instrument to measure the PPS extension.

This task is based on a vast number of neuroscientific studies on the primate brain, which reported how it represents far and near spaces differently, treating the space close to the body as distinct from farther space (for a review, see [4]). This is evident in the spatial extent of multisensory receptive fields of neurons in specific frontoparietal brain areas. These neurons respond to and integrate tactile stimulation on the body with visual or auditory information from external stimuli, but only within a limited spatial range from the body, which is considered the extent of the PPS [42,43]. Building upon this neuroscientific understanding, Serino and colleagues [4,8] developed an audio–tactile task designed to leverage these multisensory receptive fields. In this paradigm, participants are presented with a tactile stimulus and a concurrent dynamic auditory stimulation that approaches or recedes from the participant, which should be ignored. If both stimuli are presented when the auditory stimulus is perceived near the body, i.e., inside the boundary of the PPS, then this is expected to facilitate tactile processing due to the concurrent activation of the frontoparietal multisensory neurons. This facilitation should result in faster reaction times to the tactile stimulus (for behavioral findings see [44,45]). The critical space distance at which such multisensory facilitation starts happening is considered the boundary of PPS. However, another important property of this task is that, along with the PPS extensions, it also assesses the sharpness of the PPS boundary, that is, the quickness of the transition from PPS to extra-personal space [8]. By using this multisensory interaction task, several studies have reported alterations of PPS sharpness irrespectively of the alterations of PPS extent, which may indicate that the distinction between near and far space or between the self and the outer world becomes weaker [46,47,48,49].

Our study aims to explore the state effects of short-term meditation induction on both the extension and the sharpness of peripersonal space (PPS) boundaries in a group of nonmeditators. As in Nguyen et al. [33], we employed a pre–post design in which the PPS of participants was assessed before and after a 15-min guided meditation. However, differently from Nguyen et al. [33], we introduced two important elements: First, we employed a typical form of MM, namely the focused-attention meditation, while Nguyen et al. [33] used an uncoded form of meditation based on visual imagery, in which participants were asked to “imagine” body modification, specifically in terms of expansion toward the outward space. During FAM, instead, participants are asked to simply “feel” the moment-to-moment experience of body sensory modification, as related to the sensation of breathing. Second, in our study, participants performed a different task to assess PPS, i.e., the multisensory audio–tactile task (based on [8,50]). This task is widely used in the literature and, importantly, it provides two distinct parameters of PPS representation: (a) the extensions of the PPS boundary, i.e., the critical distance at which subjects’ RTs to tactile stimuli are speeded up while a concurrent dynamic sound is presented near, rather than far from, the body, and (b) the sharpness of the PPS boundary, i.e., the speed of the transition from near to far space.

We hypothesized that the 15-min FAM induction might modulate the extension of the PPS boundaries in nonmeditators, as reported by Nguyen et al. [33]. Specifically, we may expect the shrinkage of PPS boundaries, given the enhanced attentional focus on breath and internal sensations due to FAM, in line with Ardizzi and Ferri [35]. Regarding the sharpness of PPS boundaries, as far as we know, no study has investigated whether it is modified by MM. However, based on phenomenological reports on the so-called sense of boundaries reported by Lindahl and Britton [24] (see also [19,32]), we hypothesize that FAM might shallow the PPS boundaries so that the transition from PPS to extra-personal space may be less sharp, in the sense that those boundaries would tend to fading or relaxing.

## 2. Materials and Methods

### 2.1. Prospective Power Analysis

Before participant enrollment, we conducted a power analysis to assess the number of participants requested for our study. The power analysis was conducted by means of G*Power 3 software [51]. The number of participants was decided based on a medium effect size of 0.23 for a repeated-measure design with power β = 0.8 and alpha = 0.05. As output, the software indicated a sample of about 25 participants to reveal a reliable effect under the constraints indicated above.

### 2.2. Participants

Twenty-six participants took part in this study (mean age: 24.1 ± 4.02; 19 female). Each participant completed the informed consent and a demographics questionnaire, asking for age, sex, and meditation experience lifetime. Participants were blind to the research purpose. Selection criteria included not being on psychopharmacological treatment, not having a psychopathological diagnosis (e.g., depression, anxiety), and not having prior experience with meditation practices. Furthermore, participants with skin problems that could interfere with the application of electrodes to sensitive facial areas, such as the cheek, were not included. The recruitment of participants was carried out by posting advertisements at the Faculty of Psychology of the Sapienza, University of Rome. Participants did not receive any compensation or credits for their participation in this study. All participants reported having no experience in meditation. Informed written consent was obtained from all participants before the experimental session. Ethical approval was obtained from the Institutional Ethics Committee.

### 2.3. Apparatus and Stimuli

In the audio–tactile task, participants were blindfolded and seated beside a table on which the audio–tactile apparatus was mounted on, in a soundproof room. Two loudspeakers were in the room: the first one was positioned next to the participants’ left cheek (“near loudspeaker”) at an elevation of 100 cm and 5 cm distant from the participants’ left cheek; the second was far from the participants (“far loudspeaker”), with the same elevation and 100 cm distant from the near loudspeaker. Auditory stimuli from the loudspeakers were samples of pink noise (44.1 kHz), lasting 3000 ms, whose intensity was manipulated for having an approaching dynamic sound (IN sound) with exponentially rising acoustic intensity from 55 to 70 dB of Sound Pressure Level (SPL). For the IN sound, the far loudspeaker started at the maximum intensity, and its intensity decreased along the trial, with the near loudspeaker intensity gradually rising so that it gave the impression of a sound source moving from the far to the near loudspeaker. Tactile stimuli were administered on the participants’ left cheek, delivered using a constant-current electrical stimulator (DS7A, Digitimer, Hertfordshire, United Kingdom) via a neurological tip electrode. The electrical stimulus was a single, constant voltage, rectangular monophasic pulse (duration = 100 μs), whose intensity was set before the experiment, in a separate calibration session, individually for each subject to be clearly above thresholds. During the meditation session, participants were guided in a 15-min mindfulness meditation by a prerecorded male voice, which they could hear using two loudspeakers while being blindfolded and comfortably seated beside a table. The task was developed and delivered via E-prime 2.0 software.

### 2.4. Design and Procedure

**Figure 1:**
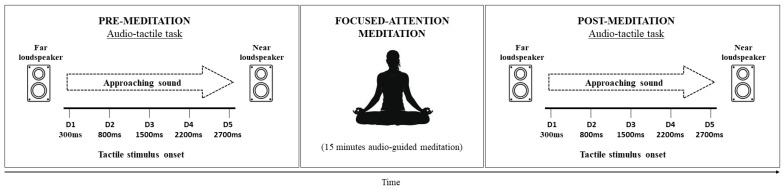
Representation of the procedure.

The procedure is represented in Figure 1. Each participant performed the audio–tactile task before and after the 15 min of guided FAM.

#### 2.4.1. The Audio–Tactile Task

The task was based on Teneggi et al. [8]. Before the experiment started, a series of 10 trials were presented, including 2 catch trials with no tactile stimulation, only auditory. Once blindfolded, participants were instructed to press the spacebar of a keyboard placed in front of them on a table every time they felt the tactile stimulus on their cheek. In the case of incorrect performance, the intensity rate was adjusted (by increasing or decreasing by 5 mA), and the procedure was repeated. The intensity range for tested participants was 60–90 mA. In each trial, the sound lasted for 3000 ms and was preceded and followed by 1000 ms of silence. Tactile stimuli were delivered at 5 different temporal delays (from T1 to T5) from the onset of the auditory stimulus as follows: T1 at 300 ms; T2 at 800 ms; T3 at 1500 ms; T4 at 2200 ms; and T5 at 2700 ms. Tactile stimuli were administered at two further temporal delays as well: T0, which preceded the onset of the sound, and T6, which followed the end of the auditory stimulus. Thus, the tactile stimulation occurred along with different perceived distances of the sound source with respect to the participant’s body. The experimental session included 62 trials, i.e., 6 target stimuli for each temporal delay (from T0 to T6), for a total of 42 trials, randomly intermingled with 20 catch trials, in which only the sound was presented. Reaction times to tactile stimuli were recorded in milliseconds as the time interval between the beginning of the trial and the participants’ response (pressing a button) at each time delay. RTs for the different temporal delays were adjusted for the time at which tactile stimulation was administered. Average RTs for each condition were then used for statistical analysis.

#### 2.4.2. Focused-Attention Meditation Session

Once blindfolded, in a soundproof room, participants had a 15-min focused-attention meditation induction based on [33]. As they were seated straight-backed, a prerecorded male voice (adapted from Yordanova et al. [37]) guided them to focus their attention on their breathing and breathing-related sensations and to sustain it over time, bringing back attention to breathing whenever they lost their focus due to their mind wandering. Before starting the FAM session, participants first read some instructions regarding the meditation practice. The instructions specified that they would be guided by a prerecorded voice to perform a meditative exercise of focused-attention meditation. Specifically, they were informed that the meditative exercise required to maintain an attentive focus to and awareness of the sensation of breathing arising from the movements of the abdomen or chest during the natural act of inhaling and exhaling. Furthermore, it was specified that during this exercise, the participant’s mind would likely start to wander into past thoughts or future projects (i.e., mind wandering), thereby losing the focus of the meditative exercise (i.e., awareness of the sensation of breathing). It was therefore specified that this mind wandering was entirely normal and part of the exercise and not to judge this activity as wrong but to notice each time they became aware of their mind wandering and consequently let go of that thought and return to the main task, bringing the focus of attention to the sensation of breathing as it exists at every moment. Once the instructions were read, the participant discussed them with the experimenter, who repeated the same instructions and provided two practical examples of mind wandering and of the returning awareness to the sensation of breathing.

During the meditation, participants followed the prerecorded voice through the 15-min meditative session, during which they were encouraged to maintain awareness of the breathing sensations and to let go of distracting thoughts or mental contents. A complete transcription of the meditation is provided in Appendix A. At the end of the meditation session, participants were asked to assess how successful they felt they had been in performing the breathing focused-attention meditation practice, responding on a Likert scale from 1 to 5, where 1 = Not at all, 2 = Slightly, 3 = Moderately, 4 = Very, 5 = Extremely.

### 2.5. Data Analysis

To investigate whether FAM modulates PPS extent and the sharpness of its boundary, RTs to tactile stimuli administered in the audio–tactile task were analyzed. First, data were filtered: we removed the trials which lacked tactile stimulation (catch trials) and in which the tactile stimuli preceded or followed the sound. As such, we considered only the trials in which both tactile and auditory stimulations were provided and which were delivered at the delays of interest: 300, 800, 1500, 2200, and 2700 ms. Further, we excluded all the trials in which subjects did not respond to the tactile stimulation. Two separate analyses were performed. Firstly, a sigmoid function was used to model the overall average RTs to tactile stimulations for each delay (from D1 to D5) and each session (pre, post) [8,52]. The equation of the sigmoid function is defined as follows, *y*(*x*) = (*y_min_* + *y_max_* ∗ *e*^(*x*−*xc*)/*b*^)/(1 + *e*^(*x*−*xc*)/*b*^), where *x* is the independent variable (delay), *y* is the dependent variable (RT), and the parameters *y_min_* and *y_max_* correspond to the saturation levels of the function. The value *x_c_* denotes the abscissa at the central point of the function, and *b* indicates the slope of the function at the central point. The authors who initially introduced the audio–tactile task [8,50,52] also advocate for employing the sigmoid function to model average RT outcomes. This recommendation leads to an optimal model for characterizing the PPS. Indeed, the parameters associated with this model enable the identification of two distinct features in the representation of the PPS: (a) the central point of the function, which represents the PPS boundary, and (b) the slope of the function at the central point, which represents the sharpness of the transition from the near to far space. Then, an ANOVA with Session (pre, post) and Delay (D1 vs. D2 vs. D3 vs. D4 vs. D5) as within-subjects factors on the average RTs (dependent variable) was conducted. Significant effects were then explored with post-hoc analysis by means of Holm-Bonferroni corrected *t*-tests.

## 3. Results

As a first step, we examined the average ratings provided by participants after the meditation session to assess their perceived performance in the breath-focused meditation practice. Participants were asked to rate their success on a Likert scale ranging from 1 to 5 in response to the question “How successful do you think you were in performing the breath-focused meditation practice?” The average reported value was 3.52, indicating that, despite being nonmeditators, they reported a more than sufficient level of proficiency in executing the meditation task.

**Figure 2:**
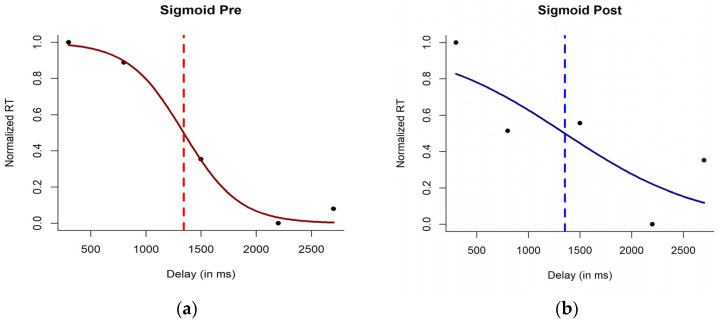
Sigmoid fitting on average normalized reaction time (RT) of the group for the audio–tactile task conducted (**a**) before (left panel) and (**b**) after the meditation session. Black dots represent actual data points averaged over all the participants. The red and blue lines represent, respectively, sigmoid fitting for the pre- and post-meditation session. The vertical dotted line in each plot represents the corresponding boundary of PPS (as *x_c_*) for that session.

Then, we modeled the overall average RTs of the group for each delay in the two sessions (pre vs. post) with the sigmoid function. We focused on the parameters x_c_ and *b* of the function, which represent the central point of the function and the slope of the function at the central point. In other words, *x_c_* is that point where RTs to tactile stimuli start being affected by the concurrent sound, thus indicating the boundary of PPS. Instead, *b* indicates the sharpness of such boundary: the lower the *b*, the higher the sharpness of the boundary. Results are reported in Figure 2 and show that before the meditative induction, the value of *x_c_* was = 1342.41, and *b* = −251.28. After the meditative induction, the value of *x_c_* was = 1353.54, and *b* = −671.84. This descriptive analysis supported a change in the shape of the PPS after the meditative session, with boundaries being less sharp and almost disappearing in the post-meditation assessment. Instead, the extension of the PPS appears to be relatively stable between the two sessions (Figure 2). Based on this analysis, we expected to find a clear transition for RTs on tactile stimuli presented with sound stimuli inside and outside the PPS boundary in the pre-meditation session but no clear transition of PPS boundaries for the post-meditation session.

We then ran an overall repeated measures ANOVA on RTs, with Delay and Session as independent variables. The ANOVA revealed a significant effect of the Delay, F_(4,100)_ = 3.99, *p* = 0.005, η_p_^2^ = 0.14, and a significant effect of Session, F_(1,25)_ = 6.52, *p* = 0.017, η_p_^2^ = 0.21, whereas the interaction Session x Delay did not reach significance, F_(4,100)_ = 0.96, *p* = 0.42, η_p_^2^ = 0.04. Although the interaction effect between Delay and Session in the overall ANOVA did not attain significance, suggesting that the changes across delays did not differ between the pre- and post-meditation sessions, the results obtained from the sigmoidal fitting support a difference in the pattern of reaction times (RTs) on stimulus delay for the two sessions. Therefore, we ran two separate one-way ANOVAs, one on the pre-meditation data and one on the post-meditation data, to better understand the pattern of results separately for each session. Each ANOVA included only Delay as the independent variable.

**Figure 3:**
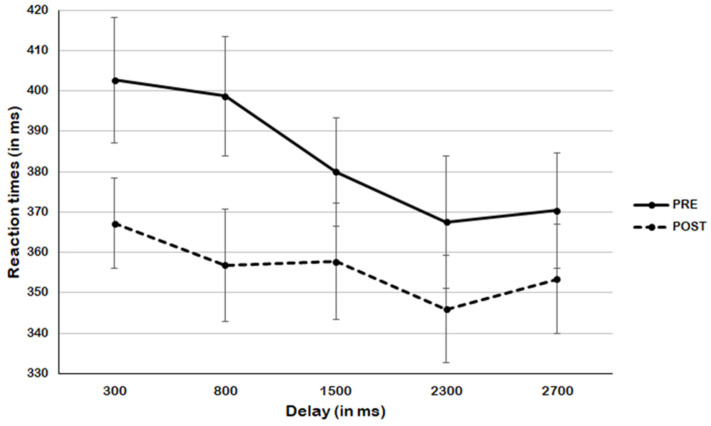
Reaction times for each delay in pre- and post-meditation sessions. All measures are reported as mean ± SEM.

The ANOVA on pre-meditation session data revealed a significant main effect of Delay, F_(4,100)_ = 4.20, *p* = 0.004, η_p_^2^ = 0.14. Holm–Bonferroni post hoc comparisons revealed that this result could be explained as a significant difference between RTs at delay 300 and both delay 2300 (*p* = 0.021, η_p_^2^ = 0.28) and 2700 ms (*p* = 0.041, η_p_^2^ = 0.25), as well as between RTs at delay 800 and 2300 ms (*p* = 0.049, η_p_^2^ = 0.23), with a trend toward significance for comparison between RTs at delay 800 and 2700 ms (*p* = 0.086, η_p_^2^ = 0.20) (see Figure 3). Overall, this analysis confirmed the pattern observed in the sigmoid modeling of the data, indicating a transition of the audio–tactile stimuli when they entered the PPS boundary at around 1500 ms. Instead, the ANOVA on the RTs after the meditation induction revealed no significant effect of Delay, F_(4,100)_ = 0.96, *p* = 0.43, η_p_^2^ = 0.04. Indeed, this again confirmed the sigmoidal fitting, indicating no clear transition for the audio–tactile stimuli presented outside and inside the boundary of the PPS.

## 4. Discussion

Peripersonal space (PPS), the functionally reachable space surrounding our body, is conceived as a representation of our bodily self and the space that delineates the separation between self and others [4]. It has been suggested that alterations in bodily representation and PPS might result from a reorganization of multisensory integration involving internal and external sensory stimuli [27,53]. Considering studies reporting modulation of self-other representations by mindfulness meditation (MM), this study investigated the state effect of mindfulness meditation on PPS representations in a group of nonmeditators. We measured the extension and sharpness of PPS boundaries using an audio–tactile multisensory integration task based on Canzoneri et al. and Teneggi et al. [8,50]. PPS measurements were taken before and after inducing a 15-min guided focused-attention meditation (FAM). Consistent with prior findings reported in a recent study by Nguyen et al. [33] in a group of novice meditators, we hypothesized a narrowing in the extension of PPS, whereas, consistent with phenomenological reports., e.g., [19,24,32], we hypothesized a dissolution of the sharpness of PPS boundaries.

Our results demonstrate that after the FAM induction, the sharpness of PPS boundaries was modified, while the extension of PPS remained unchanged. Specifically, whereas in the pre-meditation condition reaction times were faster for auditory stimuli presented nearby compared with those presented farther away, in the post-meditation condition, no differences between delays were observed. Consistent with our hypothesis, these results demonstrate a fading of the boundaries of the PPS, rather than an extension or constriction of it. Our findings thus differ from the study of Nguyen et al. [33], which reported a narrowing of the PPS boundaries. As a possible explanation for this effect, Nguyen et al. [33] referred to a study by Ardizzi and Ferri [35], which reported how higher levels of dispositional interoceptive accuracy correlate with narrower PPS boundaries. According to Nguyen et al. [33], therefore, meditation might lead to a constriction of the PPS after meditation due to increased interoception resulting from the meditation. However, the meditative induction used by Nguyen et al. [33] was primarily characterized by imaginative activity (i.e., expanded body-based imagery meditation) rather than sensory-based experience. Therefore, the focused-attention meditation practice utilized in our experiment, being more suitable to investigate the role of interoception as related to meditation, e.g., [39,54], was expected to confirm such an effect following the meditative induction.

Nevertheless, our results can also be interpreted considering the study by Ardizzi and Ferri [35]. Indeed, the authors hypothesized that increased interoceptive accuracy may play a specific role in the balance and adaptability of the PPS, and in turn of the bodily self and self–other interactions. Specifically, the authors propose that higher interoception (associated with narrowed PPS) would indeed facilitate the potential extension of PPS boundaries, as demanded by nonsocial [55,56] and social [8] circumstances. Conversely, lower interoception is associated with wider and thus less adaptable PPS boundaries. Consistent with this, irrespective of the specific extension or constriction of PPS boundaries, the observed multisensory reorganization in our study might also be attributed to the action of increased interoceptive awareness mediated by FAM practice. However, this does not exclude a coexisting effect on the perceptual reorganization of external stimuli (e.g., [12,53]). It is important to note that both studies operate under the assumption that alterations in the PPS following (or during) meditation might explain the subjective experiences reported by meditators in previous phenomenological studies. These studies reported that meditators, during meditation, undergo an experience where the perceived boundaries between the self and the surrounding world gradually dissolve, eventually leading to a complete disappearance of the separation between self and non-self. Our results seem more aligned with these phenomenological reports, suggesting that after the meditation session, instead of expanding or contracting, the boundaries of the peripersonal space become more tenuous, as if they faded away, diminishing the sense of separation between the space previously perceived as personal or extra-personal.

Moreover, it is important to note that although the distance-based zone hypothesis of PPS, where neural and behavioral responses to stimuli are amplified compared with distant stimuli, is prevalent [57,58,59,60,61,62], other alternatives have also been hypothesized. Bufacchi and Iannetti [63], for instance, challenge such binary “in-or-out” definitions based on neurophysiological and behavioral evidence. They argue that numerous neurons demonstrate graded, rather than abruptly enhanced, responses to changes in stimulus proximity [64]. Similarly, psychophysical experiments have revealed graded responses to changes in stimulus proximity [46,65,66], indicating a slow transition from the peripersonal space to far space, rather than an abrupt shift. Such an interpretation appears more aligned with the possibility of an effect on boundary representation rather than displacement of the boundaries. For instance, the dissolution of PPS boundaries has been reported by Teneggi et al. [8]. Furthermore, Graziano and Cooke [67] proposed that the boundaries of the peripersonal space (PPS) might delineate the “flight distance” or flight zone [68]. According to Hediger, the extent of the flight distance is adaptable and context-dependent, being more expansive in grazing animals compared with domesticated ones, especially in the presence of an approaching threatening stimulus. Consequently, its boundaries appear to exhibit plasticity, akin to the plastic modulation observed in PPS. Neurophysiological evidence supporting the protective role of PPS indicates that the electrical stimulation of multimodal neurons within the macaque brain, part of a frontoparietal network believed to underlie the PPS representation [5], triggers defensive-like movements directed towards nearby or approaching objects [69,70,71]. Therefore, the attention directed toward stimuli in proximity to the body appears linked to defensive behavior. Further corroboration is offered by studies demonstrating the influence of anxiety and fear on spatial representation, manifesting as an expansion of the PPS after exposure to threatening stimuli [72] or predicted by claustrophobic tendencies [73], suggesting that a larger safety zone distances hazardous stimulus. In line with these observations, mindfulness meditation, by promoting a state of equanimity toward all sensory experiences, might induce a perceptual reorganization of external and potentially threatening stimuli, reflected in the reduction in perceptual boundaries of the PPS.

Future studies within the field of mindfulness meditation may investigate the relationship between these bodily (low-level) and cognitive (high-level) changes, such as increased compassion or altruism, as reported in numerous mindfulness studies (for a review, see [74]). Some theories and research suggest that modifications in bodily representation, like perceived physical body ownership or heightened body awareness, might drive higher-level alterations, such as enhancements in prosocial capacities like empathy, e.g., [75]. Trautwein et al. [27] proposed an interaction between cognitive- and bodily-self representation, implying that changes at the conceptual level can impact bodily self-representation, and vice versa. Along these lines, it could be postulated that the social changes observed due to MM practice might arise from low-level alterations in bodily sensory self-representation. It is possible that modifications in the cognitive domain, such as empathy and compassion (noted in several MM studies), could be influenced from the bottom-up by changes in self and bodily representations. Hence, as a practice centered on bodily sensations and mental events, MM might induce a reorganization of multisensory integration of sensory stimuli from the environment, leading to alterations in bodily representation, subsequently modulating the PPS. This, in turn, could facilitate self-other integration by reducing the separation from the physical and social environment. Indeed, Tang et al. [76] demonstrated that MM practice increases thickness and activation in brain areas responsible for body representation, such as the anterior insula, as well as areas involved in complex emotional and mental state representation, like the prefrontal cortex [77].

At present, it is crucial to advance our comprehension of the relationship between low-level and high-level representations and how mindfulness appears to influence both. Even more broadly, understanding this interaction between low and high levels of self-other representations in the domain of mindfulness may have implications for addressing public health issues recently highlighted by the WHO, particularly those associated with loneliness and isolation [29,30,31,78], as well as broader social and environmental issues, such as climate change and planetary health. Preliminary studies suggest that some aspects of mindfulness and decentering are associated with broader biophilic behavior (stewardship for nature) [79,80,81,82,83]. In this context, interventions that expand or dissolve narrower senses of self could be leveraged to address pressing individual and planetary health issues.

## 5. Limitation of the Study and Future Directions

One of the primary limitations of our study is the lack of a control group. Without a control group, we cannot completely rule out the effect of confounding variables such as test–retest bias on the observed pre-post effect on the PPS. To address this issue, future studies could include a control group engaging in a similar non-meditative activity matched in duration and intensity, or even active comparative interventions hypothesized to impact different mechanisms, such as relaxation exercises. Moreover, future studies could compare different meditation styles such as loving–kindness meditation, open-monitoring meditation, or breath-focused practices (see [37,38]). Such comparative analyses hold promise for elucidating the specific underlying mechanisms driving the effects of meditation on PPS. Furthermore, another area of interest could be comparing meditation traditions that are practiced with eyes open, such as the Zen tradition, versus those with eyes closed. This line of inquiry could shed light on whether perceptual alterations in PPS are contingent upon the visual mode adopted during meditation.

Although the meditation practice included in this study should not explicitly induce a state of bodily relaxation, future studies could explore the role of relaxation practices that are associated with managing stress and sympathetic tone. It is possible that simply being more relaxed leads participants to lower their defenses (reducing the fight-or-flight state), which could alter the sharpness of the PPS [69,71]. This mechanism is well-evidenced in the literature and could be explored in future studies with simple measures of autonomic tone (e.g., heart rate variability) or biomarkers (e.g., cortisol).

Another limitation of this study is the involving of non-meditators with a very short meditative induction. Future studies could also examine the effects on PPS of longer meditation induction or training, such as the mindfulness-based stress reduction (MBSR) program. Additionally, it would be interesting to investigate the trait effect of mindfulness on PPS representation of long-term meditators vs. nonmeditators to assess how and whether prolonged meditative experience influences PPS. These studies could contribute to a deeper understanding of the mechanisms underlying the effects of meditation on PPS.

Finally, while the audio–tactile task for measuring the PPS presents several advantages, there are limitations to this paradigm that are important to consider. For example, it does not capture the influence of other important sensory modalities on the PPS representation, especially the vision. The somewhat artificial setup of the task might not fully represent the complexity of real-world PPS interactions. Further, while the task models dynamic stimuli, it does not fully capture the participant’s active movement through space, limiting insights into how the PPS adapts during locomotion or other body movements. Then, individual differences in sensory processing can lead to variability in how participants perceive audio–tactile stimuli. This variability can complicate the interpretation of results and may necessitate larger sample sizes to achieve statistically significant findings. Further, in our study, tactile stimuli were administered on the cheek (based on [8,50]). This implies that we measured head-centered PPS, aligning with the behavioral and neural identification of various body-part-centered PPS representations, e.g., [84,85,86]. To gain a deeper understanding of the mechanisms underlying this representation, future studies could measure the PPS in different body regions.

## 6. Conclusions

Our study showed that a 15-min session of (focused-attention) meditation modifies the representation of the PPS in a group of nonmeditators. Specifically, we observed that FAM leads to a fading of the boundaries of the PPS. This finding aligns with the phenomenological reports from meditators, affirming that the phenomenological experience of reduced separation between self and the external world may be related to a modulation of PPS representation. We posit that both interoceptive and exteroceptive mechanisms might be involved in this multisensory reorganization of PPS. Future studies may compare different meditation techniques to gain a better understanding of the mechanisms underlying such modulation.

## Data Availability

Raw data for this research can be shared with other researchers upon reasonable request.

## Appendix A. Transcription of the Audio-Guided Focused-Attention Meditation Practice

We practice this breath-focused meditation. A practice that can have the effect of calming and focusing the mind. So, the idea is to follow the breath, the natural flow of the breath, breathing very naturally through the nose, following one breath cycle at a time.

We can also count the breaths to support attention to the breath. Inhale, exhale, and finally count one. Inhale, exhale, and count two, and so on until reaching 5, then start again from one. And so, we follow the breath where we can feel it better.

If we feel it better in the abdomen, which expands with inhalation and contracts with exhalation, we follow it in the abdomen, or if we breathe a little higher with the chest, we follow it in the chest, so with inhalation, the chest expands, with exhalation, the chest contracts. So, the idea is to truly follow the natural breath as it is, inhaling and exhaling one breath at a time, following the sensations of the breath.

If we notice that the mind is distracted by thoughts or wandering, this is entirely normal in this practice, and gently, calmly, and without judgment, we let go of the distraction and bring attention back to the object of meditation which is the breath. And we try again to follow one breath from beginning to end, and then relax attention a little.

We resume observing another breath cycle from the beginning of inhalation, the development of inhalation, to the end of inhalation, the beginning of exhalation, the development of exhalation, and the end of exhalation, naturally each at its own pace. If it’s a short breath that’s fine, if it’s a long breath, that’s fine too. Breath, precisely, natural. We simply follow it with awareness.

Naturally in the background of awareness, there are also other things, other sensations, physical, sounds, and so on. That is entirely normal, what we highlight at the center of our attention is precisely the breath. We remember the breath. Every breath. Up to 5. Inhale and exhale, naturally count one. Inhale and exhale and count two, and so on until 5 then start again from one.

We can notice that inhalation when the air enters is a bit more energizing, and invigorating and that exhalation is more relaxing. Inhaling and exhaling relaxes the mind. Inhaling and exhaling relaxes the body. Inhaling and exhaling calms the mind. And if we notice that the mind wanders, with gentleness we simply invite attention to return to the natural breath. It’s a relaxed concentration.
